# Climate change mitigation in food systems: the environmental and health impacts of shifting towards sustainable diets, a systematic review protocol

**DOI:** 10.12688/wellcomeopenres.15618.1

**Published:** 2019-12-17

**Authors:** Stephanie Jarmul, Zara Liew, Andrew Haines, Pauline Scheelbeek

**Affiliations:** 1 Department of Population Health, London School of Hygiene & Tropical Medicine, London, WC1E 7HT, UK; 2 Centre on Climate Change and Planetary Health, London School of Hygiene & Tropical Medicine, London, WC1E 7HT, UK; 3 The Department of Public Health, Environments and Society, London School of Hygiene & Tropical Medicine, London, WC1E 7HT, UK

**Keywords:** systematic review, sustainable diets, climate change mitigation, food systems, GHG, land use, water use, health

## Abstract

Food systems contribute greatly to global climate change due to their substantial contributions to greenhouse gas emissions, water use, and resource allocation. In addition, current food systems fail to deliver healthy and sustainable foods for all, with obesity as well as undernourishment remaining a pertinent global issue. Mounting pressures such as population growth and urbanisation urge rapid and transformational adaptations in food systems to sustainably feed a growing population. Sustainable diets have been promoted as a potential climate change mitigation strategy, and are characterized by high plant based foods and reduced animal-sourced and processed foods. While the evidence base on the potential health and environmental impacts of shifts towards sustainable diets has been growing rapidly over the past decade, there has been no recent synthesis of the evidence surrounding the health and climate mitigation benefits of sustainable consumption patterns. This systematic review will synthesize the evidence of both empirical and modelling studies assessing the direct health outcomes (such as all-cause mortality and body mass index) as well as environmental impacts (greenhouse gas emissions, land use, water use etc.) of shifts towards sustainable diets. Eight literature databases will be searched to identify studies published between 1999-2019 that report both health and environmental outcomes of sustainable diets. Evidence will be mapped and subsequently analysed based on the comparability of results and reported outcomes.

## Background

Food production is a major contributor to global climate change. Agriculture alone accounts for approximately 20–25% of global greenhouse gas emissions ( Smith *et al.*, 2014) and 80% of fresh water withdrawals ( Velasco-Muñoz *et al.*, 2018) and has had predominantly negative implications for biodiversity ( Gonthier *et al.*, 2014). Rapidly changing diets, increasing international trade, and a projected global population of 9.8 billion people by 2050 ( DESA, 2017) will likely increase the contribution of food production to climate change, while climate change impacts – such as heat waves and changing precipitation patterns – form additional challenges to produce enough healthy food for the planet. While advances in agricultural technologies could play a crucial role in adapting to or tackling some of these challenges (such as improving efficiency of inputs and land use requirements), promoting sustainable dietary choice may be an effective strategy for climate change mitigation.

In the past century, there has been a global shift from ‘traditional’ diets comprising mostly plant-based and minimally processed foods, towards diets characterised by a high consumption of animal-sourced and highly processed foods ( Popkin, 2006). Recent studies have highlighted the health benefits of diets comprising reduced animal-sourced food consumption (often focussing on red and processed meat) and high levels of plant-based foods, including fruits and vegetables. These diets are not only associated with decreases in non-communicable diseases ( Krishnaswamy & Gayathri, 2018; Tokunaga *et al.*, 2012), but are also associated with lower environmental footprints ( Aleksandrowicz *et al.*, 2016; Perignon *et al.*, 2017). Shifts from ‘current’ to more ‘sustainable diets’ could therefore serve as both a climate mitigation strategy and a strategy to improve population health.

The evidence base on health co-benefits of sustainable diets has been growing rapidly with many global, regional, national and sub-national (modelling) studies estimating the potential impact of dietary change on both the environment and health. Furthermore, several global initiatives have started to shape the practicalities of ‘sustainable diets’, with the EAT-Lancet Report as one of the most recent examples ( Willett *et al.*, 2019). While studies at a global level appear to consistently have found positive impacts on population health of shifts towards more sustainable diets ( Nelson *et al.*, 2016), results from analyses at regional, national and sub-national scale could vary greatly ( Aleksandrowicz *et al.*, 2016; Springmann *et al.*, 2018). Furthermore, evidence from observational and experimental studies as well as studies simultaneously measuring environmental and health impacts of sustainable diets remains scant with no recent and comprehensive data evidence synthesis.

In this review we will provide a synthesis of the evidence around the health and environmental impacts of shifts towards more sustainable diets. In order to provide a more precise summary of the combined climate change mitigation and health impacts of sustainable diets, search terms will be optimised to capture studies reporting both health and environmental outcomes of evaluated diets/consumption patterns as well as observational and experimental studies. We will include studies from October 1999 to October 2019 in all languages (that included an abstract in English) from all geographical locations and aggregate data that meet our quality and inclusion criteria. This systematic review builds upon previous reviews ( Aleksandrowicz *et al.*, 2016 and Nelson *et al.*, 2016) but also includes additional elements such as:

1. the broadening of databases consulted (eight databases will be searched);
2. the prioritization of health as well as environmental outcomes in our search strategy;
3. the exclusion of papers defining a health outcome based on nutrients and adherence to dietary guidelines alone rather than a direct health impact; and
4. the inclusion of papers that define a change in consumption patterns as well as a particular ‘diet’ and associated environmental and health impacts.

## Objectives and research question(s)

The study objectives are to synthesise the evidence from empirical and modelling studies of the effect on 1) population health and 2) climate change mitigation of shifts towards sustainable diets.

The research question is “What are the impacts of shifts from ‘current’ to ‘sustainable’ diets on climate change mitigation and population health?”, whereby the following definitions are observed:

Population health

- Prevalence of obesity, prevalence of overweight, prevalence underweight, prevalence of nutrient deficiencies (iron, iodine, vitamin D, vitamin B12, calcium, vitamin A, zinc, magnesium)
- Risk and mortality of hypertension, stroke, ischaemic heart disease, diet related cancers (colorectal, oesophagus, stomach, lung, other), diabetes, chronic kidney disease, and other diet related chronic diseases
- All-cause and premature mortality rate and/or diet related morbidity

Climate change mitigation

- Differences in greenhouse gas emissions of sustainable diets as compared to current diets
- Differences in water requirements of sustainable diets as compared to current diets
- Differences in land requirements of sustainable diets as compared to current diets
- Differences biodiversity loss of sustainable diets as compared to current diets
- Differences in nitrogen pollution of sustainable diets as compared to current diets

Current diets

- Diets, or consumption patterns, that are representative for a defined population or sub-population measured as part of a nutritional survey, or purposively collected at baseline for an intervention study

Sustainable diets

- Diets that are found to have lower environmental impacts (greenhouse gas emissions, water footprints, impacts on biodiversity, nitrogen pollution, other) compared to current diets and include the following diets: vegan, vegetarian, flexitarian, pescatarian, high plant-based foods, low animal-sourced foods, low dairy, low meat, high fruits, high vegetables, high fruits and vegetables

## Protocol

### Search strategy

The following eight literature databases will be searched with the search concepts presented in Table 1– Table 7 and Box 1 for literature published between October 1999 and October 2019.

1) OvidSP Medline ( Table 1)
2) OvidSP Embase ( Table 2)
3) EBSCO GreenFILE ( Table 3)
4) Web of Science Core Collection ( Table 4)
5) Scopus ( Table 5)
6) OvidSP CAB Abstracts ( Table 6)
7) OvidSP AGRIS ( Box 1)
8) OvidSP Global Health ( Table 7)

**Table 1.** Search strategy for OvidSP Medline.

Search #	Search term
1	(health* OR well-being OR wellbeing).ti,ab.
2	(prevalence OR incidence OR risk OR rate OR mortality OR morbidity).ti,ab.
3	1 OR 2
4	(obesity OR malnutrition OR malnour*).ti,ab.
5	(overweight OR over-weight).ti,ab.
6	(underweight OR under-weight).ti,ab.
7	((nutrient OR iron OR iodine OR “vitamin d” OR “vitamin b12” OR calcium OR “vitamin a” OR zinc OR magnesium) adj2 (deficien* OR shortage* OR value*)).ti,ab.
8	(anemia or anaemia).ti,ab.
9	(hypertension OR “blood pressure” OR BP OR stroke).ti,ab.
10	(diabetes OR ICH OR “heart disease” OR CKD OR “kidney disease” OR chronic).ti,ab.
11	(cardiovascular OR cardio-vascular).ti,ab.
12	cancer.ti,ab.
13	((environment* OR climate*) adj5 (friendly OR sustainab* OR footprint or foot-print or "foot print" or biodivers* or nitrogen or impact* or damage* or greenhouse or land* or “land use” or water* or use* or benefit* OR implication* OR carbon)).ti,ab.
14	(vegan* or vegetarian* or flexitarian* or pescatarian* or fish* OR sea-food OR seafood).ti,ab.
15	((meat or animal-sourced or "animal sourced" or ultra-processed or "ultra processed" or UPF or dairy) adj3 (reduc* or decreas* or free)).ti,ab
16	((plant-based OR “plant based” OR plantbased OR fruit* OR vegetable* OR legume* OR nut* OR pulse*) adj3 (increas* OR higher)).ti,ab.
17	((diet* or consum* or "eating pattern" or meal* or nourish*) adj3 (current or average* or change* or shift* or choice* or scenario* or habit* or sustain*)).ti,ab.
18	4 OR 5 OR 6 OR 7 OR 8 OR 9 OR 10 OR 11 OR 12
19	3 AND 17
20	14 OR 15 OR 16 OR 17
21	13 ND 19 AND 20

**Table 2.** Search strategy for OvidSP Embase.

Search #	Search term
1	(health* OR well-being OR wellbeing).ti,ab.
2	(prevalence OR incidence OR risk OR rate OR mortality OR morbidity).ti,ab.
3	1 OR 2
4	(obesity OR malnutrition OR malnour*).ti,ab.
5	(overweight OR over-weight).ti,ab.
6	(underweight OR under-weight).ti,ab.
7	((nutrient OR iron OR iodine OR “vitamin d” OR “vitamin b12” OR calcium OR “vitamin a” OR zinc OR magnesium) adj2 (deficien* OR shortage* OR value*)).ti,ab.
8	(anemia or anaemia).ti,ab.
9	(hypertension OR “blood pressure” OR BP OR stroke).ti,ab.
10	(diabetes OR ICH OR “heart disease” OR CKD OR “kidney disease” OR chronic).ti,ab.
11	(cardiovascular OR cardio-vascular).ti,ab.
12	cancer.ti,ab.
13	((environment* OR climate*) adj5 (friendly OR sustainab* OR footprint or foot-print or "foot print" or biodivers* or nitrogen or impact* or damage* or greenhouse or land* or “land use” or water* or use* or benefit* OR implication* OR carbon)).ti,ab.
14	(vegan* or vegetarian* or flexitarian* or pescatarian* or fish* OR sea-food OR seafood).ti,ab.
15	((meat or animal-sourced or "animal sourced" or ultra-processed or "ultra processed" or UPF or dairy) adj3 (reduc* or decreas* or free)).ti,ab
16	((plant-based OR “plant based” OR plantbased OR fruit* OR vegetable* OR legume* OR nut* OR pulse*) adj3 (increas* OR higher)).ti,ab.
17	((diet* or consum* or "eating pattern" or meal* or nourish*) adj3 (current or average* or change* or shift* or choice* or scenario* or habit* or sustain*)).ti,ab.
18	4 OR 5 OR 6 OR 7 OR 8 OR 9 OR 10 OR 11 OR 12
19	3 AND 17
20	14 OR 15 OR 16 OR 17
21	13 ND 19 AND 20

**Table 3.** Search strategy for EBSCO GreenFILE.

Search #	Search term
S1	(health* OR wellbeing OR well-being)
S2	(prevalence OR incidence OR risk OR rate OR mortality OR morbidity)
S3	(obesity OR malnutrition OR malnour*)
S4	(underweight OR under-weight)
S5	(overweight OR over-weight)
S6	((nutrient OR iron OR iodine OR “vitamin D” OR “Vitamin B12” OR calcium OR “Vitamin A” OR zinc OR magnesium) N2 (deficien* OR shortage* OR value*))
S7	(anemia OR anaemia)
S8	"blood pressure”
S9	(hypertension OR stroke OR diabetes OR ICH OR chronic)
S10	”heart disease”
S11	”kidney disease”
S12	(CKD OR cardio-vascular OR cardiovascular OR BP)
S13	cancer
S14	S1 OR S2
S15	S3 OR S4 OR S5 OR S6 OR S7 OR S8 OR S9 OR S10 OR S11 OR S12 OR S13
S16	S14 AND S15
S17	((environment* OR climate*) N5 (friendly OR footprint OR foot-print OR "foot print" OR impact* OR damage* OR greenhouse or land* OR “land use” OR water* OR use* OR benefit* OR implication* OR carbon OR sustain* OR nitrogen* OR biodiverse*))
S18	(vegan* OR vegetarian* OR flexitarian* OR pescatarian* OR fish* OR sea-food OR seafood)
S19	((meat OR animal-sourced OR "animal sourced" OR ultra-processed OR "ultra processed" OR UPF OR dairy) N3 (reduc* OR decreas* OR free))
S20	((plant-based OR “plant based” OR plantbased OR fruit* OR vegetable* OR legume* OR nut* OR pulse*) N3 (increas* OR higher))
S21	((diet* OR consum* OR "eating pattern" OR meal* OR nourish*) N3 (current OR average* OR change* OR shift* OR choice* OR scenario* OR habit* OR sustain*)
S22	S18 OR S19 OR S20 OR S21
S23	S16 AND S17 AND S22

**Table 4.** Search strategy for Web of Science Core Collection.

Search #	Search Term
#20	#13 AND #14 AND #19
#19	#15 OR #16 OR #17 OR #18
#18	TS=((diet* OR consum* OR “eating pattern” OR meal* OR nourish*) near/3 (current OR average* OR change* OR shift* OR choice* OR scenario* OR habit* OR sustain*))
#17	TS=((plant-based OR fruit* OR vegetable* OR legume* OR nut* OR pulse*) near/3 (increas* OR higher))
#16	TS=((meat OR animal-sourced OR dairy OR ultra-processed OR UPF) near/3 (reduc* OR decreas* OR free ))
#15	TS=(vegan* OR vegetation* OR flexitarian* OR pescatarian* OR sea-food OR seafood OR fish*)
#14	TS=((climate OR environment*) near/5 (friendly OR footprint OR foot-print OR “foot print” OR impact* OR damage* OR greenhouse OR land* OR “land use” OR water* OR use* OR benefit* OR implication* OR carbon* OR sustain* OR biodivers* OR nitrogen))
#13	#11 AND #12
#12	#3 OR #4 OR #5 OR #6 OR #7 OR #8 OR #9 OR #10
#11	#1 OR #2
#10	TS=(CKD OR cardiovascular OR cardio-vascular OR cancer OR BP)
#9	TS=”kidney disease”
#8	TS=”heart disease”
# 7	TS=(hypertension OR stroke OR diabetes OR ICH OR chronic)
# 6	TS="blood pressure”
#5	TS=(anemia OR anaemia)
# 4	TS= ((nutrient OR iron OR iodine OR “vitamin D” OR “vitamin B12” OR calcium OR “Vitamin A” OR zinc OR magnesium) near/2 (deficien* OR shortage* OR value*))
# 3	TS=(obesity OR overweight OR over-weight OR underweight OR under-weight OR malnutrition OR malnour*)
# 2	TS=(prevalence OR incidence OR risk OR rate OR mortality or morbidity)
# 1	TS=(health* OR wellbeing OR well-being)

**Table 5.** Search strategy for Scopus.

Search term
**TITLE-ABS-KEY** (health* OR well-being OR prevalence OR incidence OR risk OR rate OR mortality OR morbidity)
**AND TITLE-ABS-KEY** (obesity OR over-weight OR under-weight OR malnutrition OR malnour* OR ((nutrient OR iron OR iodine OR “vitamin D” OR “vitamin B12” OR calcium OR “vitamin A” OR zinc OR magnesium) W/2 (deficien* OR shortage* OR value*)) OR anemia OR anaemia OR hypertension OR “blood pressure” OR BP OR stroke OR diabetes OR ICH OR chronic OR “heart disease” or CKD OR “kidney disease” OR cardio-vascular OR cancer)
**AND TITLE-ABS-KEY** ((climate* OR environment*) W/5 (friendly OR footprint OR foot-print OR impact* OR damage* OR greenhouse OR land* OR “land use” OR water* OR use* OR benefit* OR implication* OR carbon* OR sustain* OR nitrogen* OR biodivers*))
**AND TITLE-ABS-KEY** (vegan* OR vegetarian* OR flexitarian* OR pescatarian* OR seafood OR sea-food OR fish*) OR ((meat OR animal-sourced OR ultra-processed OR UPF OR dairy) W/3 (reduc* OR decreas* OR free )) OR ((plant-based OR fruit* OR vegetable* OR legume* OR nut* OR pulse*) W/3 (increas* OR higher)) OR ((diet* OR consum* OR “eating pattern” OR meal* OR nourish*) W/3 (current OR average* OR change* OR shift* OR choice* OR scenario* OR habit* OR sustain*))

**Table 6.** Search strategy for OvidSP CAB Abstracts.

Search #	Search term
1	(health* OR well-being OR wellbeing).ti,ab.
2	(prevalence OR incidence OR risk OR rate OR mortality OR morbidity).ti,ab.
3	1 OR 2
4	(obesity OR malnutrition OR malnour*).ti,ab.
5	(overweight OR over-weight).ti,ab.
6	(underweight OR under-weight).ti,ab.
7	((nutrient OR iron OR iodine OR “vitamin d” OR “vitamin b12” OR calcium OR “vitamin a” OR zinc OR magnesium) adj2 (deficien* OR shortage* OR value*)). ti,ab.
8	(anemia or anaemia).ti,ab.
9	(hypertension OR “blood pressure” OR BP OR stroke).ti,ab.
10	(diabetes OR ICH OR “heart disease” OR CKD OR “kidney disease” OR chronic).ti,ab.
11	(cardiovascular OR cardio-vascular).ti,ab.
12	cancer.ti,ab.
13	((environment* OR climate*) adj5 (friendly OR sustainab* OR footprint or foot-print or "foot print" or biodivers* or nitrogen or impact* or damage* or greenhouse or land* or “land use” or water* or use* or benefit* OR implication* OR carbon)).ti,ab.
14	(vegan* or vegetarian* or flexitarian* or pescatarian* or fish* OR sea-food OR seafood).ti,ab.
15	((meat or animal-sourced or "animal sourced" or ultra-processed or "ultra processed" or UPF or dairy) adj3 (reduc* or decreas* or free)).ti,ab
16	((plant-based OR “plant based” OR plantbased OR fruit* OR vegetable* OR legume* OR nut* OR pulse*) adj3 (increas* OR higher)).ti,ab.
17	((diet* or consum* or "eating pattern" or meal* or nourish*) adj3 (current or average* or change* or shift* or choice* or scenario* or habit* or sustain*)). ti,ab.
18	4 OR 5 OR 6 OR 7 OR 8 OR 9 OR 10 OR 11 OR 12
19	3 AND 17
20	14 OR 15 OR 16 OR 17
21	13 AND 19 AND 20

**Table 7.** Search strategy for OvidSP Global Health.

Search #	Search term
1	(health* OR well-being OR wellbeing).ti,ab.
2	(prevalence OR incidence OR risk OR rate OR mortality OR morbidity).ti,ab.
3	1 OR 2
4	(obesity OR malnutrition OR malnour*).ti,ab.
5	(overweight OR over-weight).ti,ab.
6	(underweight OR under-weight).ti,ab.
7	((nutrient OR iron OR iodine OR “vitamin d” OR “vitamin b12” OR calcium OR “vitamin a” OR zinc OR magnesium) adj2 (deficien* OR shortage* OR value*)).ti,ab.
8	(anemia or anaemia).ti,ab.
9	(hypertension OR “blood pressure” OR BP OR stroke).ti,ab.
10	(diabetes OR ICH OR “heart disease” OR CKD OR “kidney disease” OR chronic).ti,ab.
11	(cardiovascular OR cardio-vascular).ti,ab.
12	cancer.ti,ab.
13	((environment* OR climate*) adj5 (friendly OR sustainab* OR footprint or foot-print or "foot print" or biodivers* or nitrogen or impact* or damage* or greenhouse or land* or “land use” or water* or use* or benefit* OR implication* OR carbon)).ti,ab.
14	(vegan* or vegetarian* or flexitarian* or pescatarian* or fish* OR sea-food OR seafood).ti,ab.
15	((meat or animal-sourced or "animal sourced" or ultra-processed or "ultra processed" or UPF or dairy) adj3 (reduc* or decreas* or free)).ti,ab
16	((plant-based OR “plant based” OR plantbased OR fruit* OR vegetable* OR legume* OR nut* OR pulse*) adj3 (increas* OR higher)).ti,ab.
17	((diet* or consum* or "eating pattern" or meal* or nourish*) adj3 (current or average* or change* or shift* or choice* or scenario* or habit* or sustain*)). ti,ab.
18	4 OR 5 OR 6 OR 7 OR 8 OR 9 OR 10 OR 11 OR 12
19	3 AND 17
20	14 OR 15 OR 16 OR 17
21	13 AND 19 AND 20

Box 1. **Search strategy for OvidSP AGRIS**

((health* OR well-being OR prevalence OR incidence OR risk OR rate OR mortality OR morbidity) AND (((obesity OR over-weight OR under-weight OR malnutrition OR malnour*) OR ((nutrient OR iron OR iodine OR “vitamin D” OR “vitamin B12” OR calcium OR “vitamin A” OR zinc OR magnesium) NEAR/2 (deficien* OR shortage* OR value*)) OR anemia OR anaemia OR hypertension OR “blood pressure” OR BP OR stroke OR diabetes OR ICH OR chronic OR “heart disease” or CKD OR “kidney disease” OR cardio-vascular OR cancer))) AND ((climate* OR environment*) NEAR/3 (friendly OR footprint OR foot-print OR impact* OR damage* OR greenhouse OR land* OR “land use” OR water* OR use* OR benefit* OR implication* OR carbon* OR sustain* OR nitrogen* OR biodivers*)) AND (((vegan* OR vegetarian* OR flexitarian* OR pescatarian* OR seafood OR sea-food OR fish*) OR ((meat OR animal-sourced OR ultra-processed OR UPF OR dairy) NEAR/3 (reduc* OR decreas* OR free )) OR ((plant-based OR fruit* OR vegetable* OR legume* OR nut* OR pulse*) NEAR/3 (increas* OR higher)) OR ((diet* OR consum* OR “eating pattern” OR meal* OR nourish*) NEAR/3 (current OR average* OR change* OR shift* OR choice* OR scenario* OR habit* OR sustain*))))

### Inclusion criteria

We will include peer reviewed papers – including observational, experimental and modelling studies – that cover any form of dietary shift and associated health and environmental impacts. The specific types of diets and health impacts that will be included are outlined above.

Papers in all languages (that included an abstract in English) and geographic regions will be included; where necessary, translators will be sought for the data extraction of papers in languages not spoken within the research team.

### Exclusion criteria

Papers will be excluded from review if:

- they are not relevant to the research question; or
- are review articles with no original data presented; or
- include only a description of health OR environmental outcomes, rather than both; or
- did not include baseline dietary data or a comparison population to indicate a change or “shift” from one diet to another; or
- the full texts were unobtainable after contacting the authors.

## Quality assessment and risk of bias

Study quality and potential bias will be assessed for each paper that has been selected after full-text screening. The quality criteria described in Table 8 and Table 9 will be considered for interventional/observational studies and modelling studies, respectively, and have been adapted from the CASP randomized control trial checklist ( CASP, 2018) as well as the Van Voorn checklist for modelling studies ( Van Voorn *et al.*, 2016). Studies will be ranked either low, high, or unclear for each criteria, and any papers with more than three scores of ‘high’ and/or presenting insufficient data to support the findings will be excluded from further synthesis. The quality assessment/risk of bias review will be done by the first reviewer (SJ) and a second reviewer (ZL) will independently assess 100% of the full texts included.

**Table 8.** Quality criteria for intervention/observational studies.

#	Criterion description	Issues considered
1.	Clear study description	• Did the authors provide a clear description of the dietary status/ change evaluated? • Did the authors provide a clear description of the health impacts evaluated? • Was the link with climate change mitigation and/or other environmental impacts well described? • Did the authors give a clear justification of study in a particular area – including a description of current/baseline and ‘more sustainable’ diets?
2.	Appropriate comparison group/situation	• Were the health impacts of more sustainable diets compared to an appropriate and comparable baseline group/situation? • Were inclusion/exclusion criteria of the participants clearly defined?
3.	Realistic exposure levels	• Were proposed dietary changes realistic in a specific time frame for the described context (i.e. the exposure is sufficient to develop an exposure-response estimate and there is appropriate temporality between exposure and outcome)?
4.	Clear methods description	• Did the authors clearly describe the methods used to characterize and evaluate both ‘current/ baseline’ and ‘more sustainable’ diets? • Were the methods applied to measure health and environmental impacts of evaluated diets clearly described?
5.	Rigorous and clearly described analysis	• Are sufficient data presented to support the findings? • Were analyses described in detail? • Did the researchers critically examine their potential bias and influence during measurement, analysis and selection of data for presentation?
6.	Precision of measure of effect	• How sure are we about the (causal) effect of the exposure? (using Bradford Hill) • What are the confidence limits? • Were the observed associations statistically significant?

**Table 9.** Quality criteria for modelling studies.

#	Criterion description	Issues considered
1.	Clear study description	• Did the authors provide a clear description of the dietary status/ change evaluated? • Did the authors provide a clear description of the health impacts evaluated? • Was the link with climate change mitigation and/or other environmental impacts well described? • Did the authors give a clear justification of study in a particular area – including a description of current diets?
2.	Appropriate comparison group/situation	• Were the health and/or environmental impacts of more sustainable diets compared to an appropriate and comparable baseline group/situation?
3.	Model validity/credibility	• Have the process of internal verification and its results been documented in detail? • Is there a clear description and/or justification of the assumptions, simplifications, and limitations of the model?
4.	Model suitability	• Were appropriate studies and/or data used to build the model? • Was the choice of model appropriate for the study question?
5.	Rigorous and clearly described analysis	• Are sufficient data presented to support the findings? • Were analyses described in detail? • Did the researchers critically examine their potential bias and influence during the analysis and selection of data for presentation/modelling?
6.	Precision of measure of effect	• What were the assumptions of the model? • What are the confidence limits? • Were the confidence limits statistically significant?

## Data management and extraction

A database with all search results will be developed using EndNote, comprising the identified studies after the initial search of all databases. Experts will be contacted and bibliographies of relevant papers will be searched for additional research papers that may have not been included in our initial database. Duplicates will be removed by using referencing software, as well as manual screening of titles. Subsequently all titles and abstracts will be double-screened by two researchers (SJ and ZL). Full papers of selected abstracts that meet the inclusion criteria outlined above will also be screened by two researchers to identify papers relevant to the research objectives of this study. In case of discrepancies, a third researcher (PS) will read the abstracts and/or full-texts and provide a decision on the in- or exclusion of specific papers to reduce the probability of reviewer bias.

Data will be extracted independently for details on three variables and initially summarized in Microsoft Excel:

1) Type of dietary change (i.e. shift toward vegetarian, flexitarian, increased or decreased animal-sourced food consumption). This will include the authors’ definition of the sustainable diet or dietary shift/comparison evaluated in the study, as well as a detailed description of the composition of the diets and the variation within the population. Furthermore, data on the ‘baseline’ or current diet will be collected.
2) Data on health outcomes reported in various formats including mortality, prevalence, incidence, risk, rate, or their derivatives (such as years of life lost, survival rate, and rate ratios) will be extracted from the included papers.
3) A description of the research context and anticipated climate impact of evaluated dietary shifts will be documented, including the geographical location of the study, environmental conditions for domestic food production, climate change vulnerability, water/land use, and any other contextual factors that are relevant for consumption patterns and public health in light of climate change mitigation.

## Data synthesis

Data synthesis will be conducted by the first author (SJ) and reviewed by other contributors (ZL, AH, PS)

### Evidence mapping

Given the highly diverse nature of sustainable diets we do not anticipate to perform any meta-analytical analyses, but will aim at mapping the identified literature in aggregates of specific dietary shifts (e.g. more plant based, more fruits, more vegetables will be combined), specific health outcomes (e.g. energy related outcomes such as obesity, overweight, underweight will be combined as well as nutritional quality related outcomes including all nutrient deficiencies), and environmental impact (greenhouse gas emissions, land/water use). The direction of impact (positive or negative health impact) of each of the papers by dietary aggregate will be reported and where possible graphically displayed.

### Data analysis

In case of enough quantitative data in the same ‘exposure’ and ‘outcome’ aggregate, we will explore the possibilities of quantitative pooled analyses and develop data visualisation via graphs and figures, whereby data will be presented in their absolute figures (i.e. no standardisation will be performed). Bias and the strength of the body of evidence will be assessed using quality criteria adapted from the CASP randomized control trial checklist as well as the Van Voorn checklist for modelling studies, which are further explained in Table 8 and Table 9.

### Sources of bias

Reviewer bias: Inclusion and exclusion criteria may be interpreted differently; therefore, a third reviewer will be identified if discrepancies arise.

Publication bias: If a quantitative pooled analysis is conducted, publication bias will be assessed to indicate the credibility of the results. If this is infeasible due to study heterogeneity, then lack of ability to estimate publication bias will be described as a limitation of the study in the final report.

Inconsistent outcome definitions and methods: The description and measurement of diets and consumption patterns may vary greatly between each study. Furthermore, human health and environmental parameters reported may differ by outcomes assessed and temporality. These differences will be carefully considered prior to data synthesis.

### Outputs

This review will assess population health and climate mitigation impacts of shifts toward more sustainable diets for all available geographic locations. Results of the analysis will map and/or synthesize evidence of health and environmental benefits of sustainable diets as well as help to identify gaps in the literature and inform policy decisions around supporting sustainable diets as a potential climate change mitigation strategy. Expected outputs include a comprehensive literature database, evidence mapping and/or synthesized analysis summarizing results on the environmental and health impacts of sustainable diets.

## Study status

The study protocol and search strategy have been completed; as of publication, searching has not yet begun.

## Data availability

### Underlying data

No data are associated with this article.

### Reporting guidelines

Figshare: Climate change mitigation in food systems: the environmental and health impacts of shifting towards sustainable diets, a systematic review protocol PRISMA Checklist. https://doi.org/10.6084/m9.figshare.11316593.v1 ( Jarmul *et al.*, 2019)

Data are available under the terms of the Creative Commons Zero "No rights reserved" data waiver (CC0).
