# Supplementary material for: Climate change mitigation in food systems: the environmental and health impacts of shifting towards sustainable diets, a systematic review protocol
Source: Wellcome Open Res. 2019 Dec 17;4:205. [Version 1] doi: 10.12688/wellcomeopenres.15618.1 (PMC7029755; doi:10.12688/wellcomeopenres.15618.1)
Supplement: Peer review history

# Peer review: Reviewer response for version 1

**DOI:** 10.21956/wellcomeopenres.17110.r37423

**Published:** 2020-02-18

**Authors:** Elinor Hallström

**Affiliations:** 1 Department of Agrifood and Bioscience, RISE- Research Institutes of Sweden, Gothenburg, Sweden

Thank you for the opportunity to review this interesting paper. The protocol is ambitious, thoroughly described and will provide interesting results. My main comment is that the objectives for which environmental effects that will be covered in the paper needs to be clarified. To further improve the paper I ask you to consider the following comments:

**1. Abstract**

 Consider revising the first sentence “Food systems contribute greatly to global climate change due to their substantial contributions to greenhouse gas emissions, water use, and resource allocation”. I suggest that you write “food systems contribute greatly to global environmental pressure”, since water use has no direct linkage to global climate change.

**2. Abstract**

 Consider revising the sentence “Sustainable diets have been promoted as a potential climate change mitigation strategy and are characterized by high plant based foods and reduced animal-sourced and processed foods.”

 I suggest that you revise the meaning to “limited amounts of animal sourced and processed foods”. In many populations of the world, intake levels of animal sourced food are low and are a very important source of nutrition. For animal-based foods, current intake levels in the population targeted have to be considered in order to provide dietary recommendations that are based on a holistic sustainability perspective including both environmental and health aspects. 

**3. Background**

 Consider revising the sentence “While advances in agricultural technologies could play a crucial role in adapting to or tackling some of these challenges (such as improving efficiency of inputs and land use requirements), promoting sustainable dietary choice may be an effective strategy for climate change mitigation.”

 Since previous reviews have documented a large potential for dietary change to reduce climate impact of the food system, I suggest that this sentence is revised to: “While advances in agricultural technologies could play a crucial role in adapting to or tackling some of these challenges (such as improving efficiency of inputs and land use requirements), promoting sustainable dietary choice has been identified as an effective strategy for climate change mitigation".

**4. Background**

 Consider revising the sentence “In order to provide a more precise summary of the combined climate change mitigation and health impacts of sustainable diets, search terms will be optimised to capture studies reporting both health and environmental outcomes of evaluated diets/consumption patterns as well as observational and experimental studies.”

 It is not clear if the aim of this study is to include studies analyzing diets impact on climate change or if it the aim is to analyze several environmental impact categories apart from climate change. Please revise the text to clarify this.   

**5. Background**

 “This systematic review builds upon previous reviews (Aleksandrowicz *et al.*, 2016 and Nelson *et al.*, 2016)”. Consider adding other relevant reviews focusing on dietary change and its impact on environmental and health effects, examples are provided below ^1^^-^^3^.

**6. Objectives and research questions**

 The objectives described in the article are not the same as in the abstract. Please clarify if the objectives are to study effects on climate change or as stated in the abstract to study as environmental impacts including greenhouse gas emissions, land use, water use etc. of shifts towards sustainable diets.

**7. Objectives and research questions**

 Consider revising the definition of climate change mitigation. Water and land requirement, biodiversity and nitrogen pollution can in my opinion not be defined as climate change mitigation. Please revise the sentence to “environmental impact” or another term that fits better with the included environmental indicators. 

**8. Search strategy**

 I suggest that keywords or examples of search terms used in the literature search are summarized in the main article and that Tables 1-7 are placed in an appendix. I would also recommend that the format of these tables is improved. Several tables (e.g. Table 1 and 2) provide the exact the same information and could be combined. Some of the tables provide the search numbers from top to bottom (1, 2, 3, 6, 7) whereas Table 4 provides the numbers from bottom to top

**9. Exclusion criteria**

 Please specify if the analysis is limited to include complete diets or if meals and individual food products/groups also are included in the study.

**10. Box 1**

 I don't find the format of Box 1 very illustrative and would recommend that the search strategy is described in a table, in a similar way as in Tables 1-7.

**11. Data synthesis**

 I am missing a description of if or how environmental impact data found in the literature will be modified in order to be comparable (e.g. harmonization of functional units, system boundaries etc.).

**12. Outputs**

 Could you please clarify which environmental categories you are aiming to cover. In the outputs section only climate mitigation is mentioned but in other sections other environmental impact categories are described, such as land and water use.

I confirm that I have read this submission and believe that I have an appropriate level of expertise to confirm that it is of an acceptable scientific standard, however I have significant reservations, as outlined above.

## References

[1] : Vegetarian Diets: Planetary Health and Its Alignment with Human Health. *Adv Nutr*.2019;10(Supplement_4) : 10.1093/advances/nmz019 S380-S388 10.1093/advances/nmz019 31728487PMC6855976

[2] : Environmental impact of dietary change: a systematic review. *Journal of Cleaner Production*.2015;91: 10.1016/j.jclepro.2014.12.008 1-11 10.1016/j.jclepro.2014.12.008

[3] : The impact of nutritional choices on global warming and policy implications: examining the link between dietary choices and greenhouse gas emissions. *Energy and Emission Control Technologies*.2014; 10.2147/EECT.S58518 10.2147/EECT.S58518

# Peer review: Reviewer response for version 1

**DOI:** 10.21956/wellcomeopenres.17110.r37426

**Published:** 2020-01-20

**Authors:** Sander Biesbroek

**Affiliations:** 1 Wageningen University & Research, Wageningen, The Netherlands

The work provided clearly illustrates how thoroughly the researchers detailed their plan to create a synthesis of the evidence surrounding the health and climate benefits of sustainable dietary patterns.

 I have some small comments for the authors.

 Overall: How do you intend to take regional differences in health and sustainability effects of dietary changes into account? For example, in some nutrient deficient regions an increase in meat/dairy consumption will likely add to a healthy diet (though at expense of sustainability of course). Also what about specific sub-populations with increased protein (quality and quantity) requirements, for example children or the elderly?

1. In the abstract ‘sustainable consumption patterns’ is mentioned. Although clear from the rest of the abstract that you aim to investigate dietary shift, consumption patterns as such also entail other consumptions.
2. Background, first paragraph, you mention the challenge to ‘produce enough healthy food for the planet’. The challenge will be to produce enough food and then secondly preferably healthy foods. The paragraph ends with ‘promoting ...mitigation’. I would also add that dietary changes are something consumers can directly act on themselves. 
3. Of the included addition elements, number 3 excluded nutrients as a health outcome. However, under the definition of health, nutrient deficiencies are listed.
4. Under the definition of climate change mitigation land use requirement is listed. Will this also include (expected) land use change?
5. Definition of sustainable diets: space between vegetarian, flexitarian.
6. Table 3. Other tables list 1-21 without the S in front. Table 4 is 20-1 (reversed) and with #.
7. ‘Quality assessment and risk of bias section': three or more scores on ‘high’ then papers will be excluded from the analysis. Should this not be ‘low’?

I confirm that I have read this submission and believe that I have an appropriate level of expertise to confirm that it is of an acceptable scientific standard.

# Peer review: Reviewer response for version 1

**DOI:** 10.21956/wellcomeopenres.17110.r37425

**Published:** 2019-12-27

**Authors:** Aline Martins de Carvalho

**Affiliations:** 1 Nutritional Sciences Department, School of Public Health, University of Michigan, Ann Arbor, MI, USA; 2 Department of Nutrition, School of Public Health, University of Sao Paulo, São Paulo, Brazil

It was a pleasure to review your paper. Congratulations for your work!

 In my opinion, it is mostly clear and the methods are adequate.

 I have just some questions that I would like to hear from you: how many papers do you think you will include in the final analyses? I ask that because you have tight criteria, so do you think you will have enough data to run your final analyses?

 Have you thought in adding some kind of flexibility in the inclusion criteria in order to have more data?  

 Another concern is: how will you consider the different metrics and methods from different studies and how will you analyze them together? How will it be addressed, for example, food patterns measured using 24-hour dietary recalls and Food Frequency Questionnaires from different countries, with different portion size references, different nutritional references, etc? The same thing for environmental and health measurements, how will they be addressed together?

 I believe you must add a paragraph describing with more details how you will address these differences and analyze the data.

 I'm looking forward to hearing from you.

I confirm that I have read this submission and believe that I have an appropriate level of expertise to confirm that it is of an acceptable scientific standard, however I have significant reservations, as outlined above.
